# Understanding Reproductive Aging in Wildlife to Improve Animal Conservation and Human Reproductive Health

**DOI:** 10.3389/fcell.2021.680471

**Published:** 2021-05-19

**Authors:** Pierre Comizzoli, Mary Ann Ottinger

**Affiliations:** ^1^Smithsonian Conservation Biology Institute, National Zoological Park, Washington, DC, United States; ^2^Department of Biology and Biochemistry, University of Houston, Houston, TX, United States

**Keywords:** wildlife, reproduction, fertility, aging, animal models

## Abstract

Similar to humans and laboratory animals, reproductive aging is observed in wild species-from small invertebrates to large mammals. Aging issues are also prevalent in rare and endangered species under human care as their life expectancy is longer than in the wild. The objectives of this review are to (1) present conserved as well as distinctive traits of reproductive aging in different wild animal species (2) highlight the value of comparative studies to address aging issues in conservation breeding as well as in human reproductive medicine, and (3) suggest next steps forward in that research area. From social insects to mega-vertebrates, reproductive aging studies as well as observations in the wild or in breeding centers often remain at the physiological or organismal scale (senescence) rather than at the germ cell level. Overall, multiple traits are conserved across very different species (depletion of the ovarian reserve or no decline in testicular functions), but unique features also exist (endless reproductive life or unaltered quality of germ cells). There is a broad consensus about the need to fill research gaps because many cellular and molecular processes during reproductive aging remain undescribed. More research in male aging is particularly needed across all species. Furthermore, studies on reproductive aging of target species in their natural habitat (sentinel species) are crucial to define more accurate reproductive indicators relevant to other species, including humans, sharing the same environment. Wild species can significantly contribute to our general knowledge of a crucial phenomenon and provide new approaches to extend the reproductive lifespan.

## Introduction

Reproductive aging is a process involving a combination of multiple intrinsic and extrinsic factors affecting the whole organism and more directly the reproductive organs as well as the quality of germ cells. Reproductive aging in human and laboratory animals has been studied for decades at the physiological, hormonal, cellular, and molecular levels. As gamete quality declines more rapidly in women than in men, reproductive aging leads to infertility, increased miscarriages, and birth defects (vom Saal et al., 1994; Sharma et al., 2015; Duncan and Gerton, 2018; Marshall and Rivera, 2018; Quesada-Candela et al., 2021). The menopause is the last menstrual cycle in women and other primates, which may or may not be ovulatory. Fertility is typically lost before menopause (vom Saal et al., 1994; Wu et al., 2010). While there is a depletion of ovarian reserve, there is no comparable loss in testicular germ cells. Research in aging has identified key evolutionarily conserved lifespan-mediating genetic pathways (Partridge, 2010). However, mechanisms mediating reproductive senescence and somatic lifespan in males and females remain limited. Aging of the reproductive organs and functions can occur before aging of the entire organism; this can lead to several months or years of post-reproductive life in different species (Ellison and Ottinger, 2014). On the other hand, in addition to the natural aging of the organism, environmental factors (such as, endocrine disruptors, nutrition, or stress) can impact and even accelerate reproductive aging (Ottinger, 2010).

Comparative studies in multiple animal models have been essential to improve our understanding of aging mechanisms and to develop mitigation strategies. Invertebrate and vertebrate models, including worms, birds, and mammals have provided organisms for the study of reproductive aging (Packer et al., 1998; Tatar, 2010; Jones et al., 2014). Interestingly, some processes of reproductive aging are common among species (Ottinger, 2010). For instance, germline aging observed in *Caenorhabditis elegans and Drosophila melanogaster* has conserved properties in the process of mammalian ovarian aging (Tatar, 2010). Recent studies of the mouse epigenome (i.e., global and imprinted DNA methylation, histone modifications, and epigenetic modifiers) have demonstrated its implication in physiological aging of gametes and embryos (Marshall and Rivera, 2018). In addition, maternal age impacts uterine adaptability to pregnancy and reproductive success in laboratory mice (Woods et al., 2017).

In livestock species, similar to humans, decreasing levels of anti-Mullerian hormone (AMH) during aging are associated with declining reproductive performance. This can be used as a predictor of fertility longevity in cattle; however, it still remains largely unexplored in sheep and other farm species (Mossa et al., 2017). Domestic horses can also be considered as good model for reproductive aging processes because mechanisms have been well described in mares and stallions (Turner, 2019; Carnevale et al., 2020).

In laboratory and domestic animal models, some aspects of reproductive aging have received more attention in females than males (especially about hormone levels and germ cell production). For instance, aging in the drosophila model still remains understudied in males. However, recent reports have shown that, not only sperm, but also seminal fluid are both qualitatively and quantitatively affected by age with each component making distinct contributions to declining reproductive performance in older males (Sepil et al., 2020). Studies conducted in Japanese quail (*Coturnix japonica*) males demonstrated that similar to mammals, plasma androgen levels decline gradually, being accompanied by a loss of reproductive behavior (Ottinger, 2010). Interestingly, testosterone treatment can restore reproduction in aging male quail while it is not effective in primates (Ottinger, 2010).

In addition to existing animal models, there is a need for more comparative studies on other species with different sizes, anatomies, physiologies, and life expectancies to improve our understanding of reproductive aging. The huge diversity in anatomy and reproductive physiology observed in wildlife therefore is an untapped resource for aging research (Comizzoli et al., 2018; Comizzoli and Holt, 2019). There are striking differences in reproductive aging going from semelparity (rapid senescence and death after a first breeding season like in salmonids or marsupial mouse, *Antechinus sp.*) to negligible reproductive senescence (naked mole rats, *Heterocephalus glaber*) (Edrey et al., 2011). Even though common species within a given family can often serve as models for the wild counterparts, reproductive traits are sometimes not related to taxonomy, even for closely related species (Cohen, 2018). This is why we need to keep adding many different species to our datasets. Studies in wild species (in their natural habitats or in conservation breeding centers) are invaluable because we can learn about unique adaptations especially in long-lived species, which will not only improve conservation efforts, but also provide new comparative models for other domestic species, and importantly for humans.

The objectives of this review are to (1) present conserved as well as distinctive traits of reproductive aging in different wild animal species (2) highlight the value of comparative studies to address aging issues in conservation breeding as well as in human reproductive medicine, and (3) suggest next steps forward in research.

## Lessons From Reproductive Aging in Wild Mammalian Species

The study of reproductive senescence in free-ranging populations is invaluable as it allows to better understand fundamental aspects, such as mate choice, sperm competition, or environmental impact. Furthermore, short- and long-lived wild species exhibit unique characteristics that inform our understanding of aging processes. An overview of the impact of aging on reproductive traits (including some observed in humans) in selected wild mammalian species (from rodents to non-human primates) is presented in Table 1. First, it is clear that there is still a research gap to be filled in order to have a better understanding of reproductive aging across mammalian species. However, many existing reports already highlight striking commonalities and differences between species when looking at reproductive traits known to be also affected by aging in humans. The following examples illustrate the diversity among wild mammalian species.

**TABLE 1:**
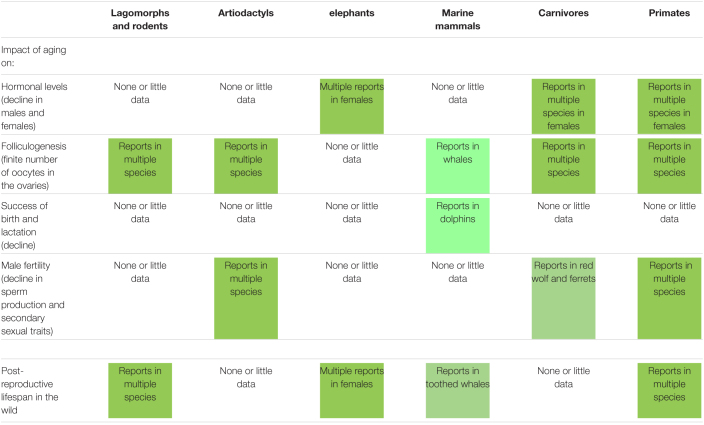
Impact of aging on different reproductive traits (including some observed in humans) and occurrence of post-reproductive lifespan in selected wild mammalian species.

*Shading indicates multiple data reports available (darker color) and moderate amount of available data (lighter color).*

In carnivores like the European badger (*Meles meles*), there is strong evidence for a gradual decline in sex-steroid levels with age in both sexes, which is even followed by over 2 years of post-reproductive life (Sugianto et al., 2020). Interestingly, this hormonal decline is not observed in aging female cheetahs (*Acinonyx jubatus*) after the age of 9, which is even older than the average life expectancy (Crosier et al., 2011).

Surprisingly, aging red deer (*Cervus elaphus*) stags show a faster decline in annual breeding success than females that went through successive gestations and lactations (Nussey et al., 2009; Bender and Piasecke, 2019). In wild red wolves (*Canis rufus*), reproductive aging in males also is observed while there is no evidence for reproductive aging in wild female wolves (Sparkman et al., 2017).

In bighorn sheep (*Ovis canadensis*), there is no consequence of early life reproduction; however, for a given number of offspring, females that weaned more sons than daughters experience earlier senescence (Douhard et al., 2020). Offspring sex ratio could help explain among-individual variation in senescence rates, perhaps in other species, including humans.

Reproductive senescence in female non-human primates involves the depletion of the ovarian reserves similar to humans (Walker et al., 2009). However, in wild chimpanzees (*Pan troglodytes*), females with high number of years spent gestating and lactating during their reproductive lives have a delayed ovulatory attrition (Atsalis and Videan, 2009). Studies in wild baboons (*Papio* sp.) also suggested that reproductive senescence occurs later in females than males (Altmann et al., 2010).

Advanced maternal age also affects the capacity to provision and rear surviving offspring. In wild populations of bottlenose dolphins (*Tursiops aduncus*), calf survival decreases with maternal age because of less maternal investment (Karniski et al., 2018).

Long-lived species such as elephants, whales and primates exhibit extended post-fertile survival compared to species with shorter lifespans. In wild African elephants (*Loxondonta africana*), although reproduction does not entirely cease until the age of 65 year old, females have a relatively long post-reproductive life (>15 years; Lee et al., 2016). Interestingly, presence of mothers and grandmothers in the group improves daughter’s reproduction (Lee et al., 2016). Note that post-reproductive life also is reported in a few species of toothed whales (Ellis et al., 2018); however, this is an uncommon trait in mammalian species compared to humans (Ellison and Ottinger, 2014).

There is no apparent decline in fertility with age in very few mammalian species, such as the tundra vole (*Microtus oeconomus*) (Cohen, 2018) or the famous female naked mole rat (*Heterocephalus glaber*) (Edrey et al., 2011; Place et al., 2021).

Lastly, in terms of extrinsic factors influencing reproductive aging, the environment plays a non-negligible role. Interestingly, in the Eurasian beaver (*Castor fiber*), earlier breeding opportunities lead to earlier senescence through resource-dependent mechanisms (Campbell et al., 2017). This clearly shows that reproductive lifespan is determined by external factors, including resource availability, in addition to reproductive strategies.

Overall, there are considerable variations among species because the correlation between age and fertility is not linear (Jones et al., 2014). While some species can be used as models for closely related species, there are often exceptions confirming the rule. In humans, reproduction mainly occurs at younger adult ages. Although more spread over the lifespan, it is similar in species like killer whales (*Orcinus orca*), chimpanzees (*Pan troglodytes*), and chamois (*Rupicapra rupicapra*). Notably, some species rather show an increase in fertility as they age (tundra voles, *Microtus oeconomus*) (Jones et al., 2014). As seen above, some patterns of reproductive aging are distinct in human compared to primates. Reproductive senescence in midlife, although apparent in natural-fertility, natural-mortality populations of humans, is generally absent in other primates (Alberts et al., 2013). Lastly, aging in females is more prevalent than in males that have continuous spermatogenesis at advanced ages in many species.

## Lessons From Reproductive Aging in Wild Non-Mammalian Species

An overview of the knowledge about the impact of aging on reproductive traits (including some observed in humans) in selected wild non-mammalian species (from corals to birds) is presented in Table 2. As observed in mammalian species, there is sometimes no relation between taxonomy and reproductive aging observations. Overall, many knowledge gaps have to be filled regarding the impact of aging on hormonal levels, number of oocytes in the ovaries, male aging (primary and secondary traits), and post-reproductive lifespan (Ellison and Ottinger, 2014). Nevertheless, current reports in non-mammalian species are highly informative and may contribute to our overall understanding of reproductive aging.

**TABLE 2:**
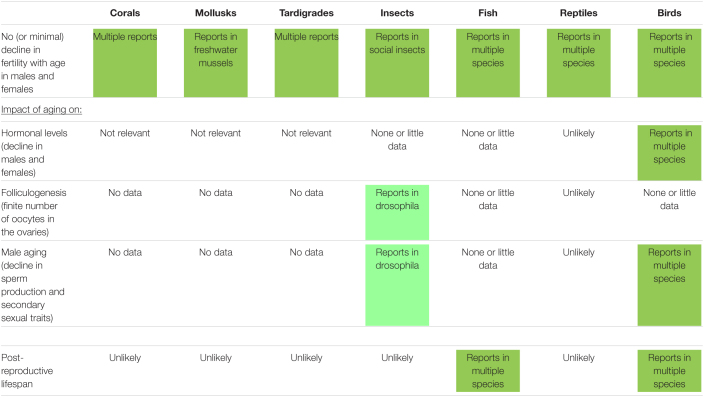
Impact of aging on different reproductive traits (including some observed in humans) and occurrence of post-reproductive lifespan in selected wild non-mammalian species.

*Shading indicates multiple data reports available (darker color) and moderate amount of available data (lighter color).*

While it is not so common in mammalian species, the absence of fertility decline with aging is reported in many different non-mammalian species from corals to birds (Nisbet et al., 1999; Bythell et al., 2018; Cohen, 2018; Table 2). Species having a sustained fertility can be very diverse like different species of fresh water mussels (Haag and Staton, 2003), tardigrades (Tsujimoto et al., 2016), queens in social insects like ants and bees (Heinze and Schrempf, 2008), fish (Greenland sharks, *Somniosus microcephalus*), reptiles (freshwater crocodile, *Crocodylus johnsoni*), and some birds (Southern fulmars, *Fulmarus glacialoides*; or Alpine swift, *Tachymarptis melba*) (Cohen, 2018).

As seen above in some wild mammals, insects like field crickets (*Gryllus campestris*) investing more in early reproduction may senesce faster and die younger (Rodríguez-Muñoz et al., 2019). In wild blue-throated warblers (*Setophaga caerulescens*) older males tend to have longer sperm cells, which may be advantageous in post-copulatory sexual selection (Cramer et al., 2020). While male aging is reported in some bird species (Lemaître and Gaillard, 2017), changes in sperm morphology with age are not observed in other birds, though they have been observed in insects and fishes (Cramer et al., 2020).

Post-reproductive lifespan does not appear to be common in non-mammalian species with only few reports in fish (herrings, *Clupea harengus*) (Benoît et al., 2020) or birds (Bali mynahs, *Leucopsar rothschildi*) (Jones et al., 2014).

## Lessons From Reproductive Aging in Conservation Breeding (*Ex Situ*)

Zoo animal populations represent an ideal model to study reproductive aging process since lifespan is frequently longer than in the wild, so repeated observations under controlled conditions are possible. Reproductive aging also represents a major issue in conservation breeding, especially for genetically valuable individuals that are too often not reproducing (Hermes et al., 2004; Crosier et al., 2020).

Research on naked mole rat (*Heterocephalus glaber*) colonies has generated intriguing data. Unusually large ovarian reserve in naked-mole rat provides one mechanism to account for this species’ protracted fertility. However, whether germ cell nests in adult ovaries contribute to the long reproductive lifespan remains to be determined (Place et al., 2021).

In terms of male aging, the black-footed ferret (*Mustela nigripes*), has revealed that even modest aging decreases sperm production and libido while abnormal sperm number increases (Wolf et al., 2000). This is one of the few examples of rapid reproductive aging in males. In contrast, recent studies in binturong (*Arctictis binturong*) show that males of advanced ages still produce good quality semen (Zainuddin et al., 2021).

Prolonged exposure to endogenous sex steroids and long stretches of non-reproductive periods correlate with early reproductive aging and pathologies in females. For instance, this is observed in nulliparous females in Asian or African elephants (*Elephas maximus*, *Loxodonta africana*) and cheetahs (*Acinonyx jubatus*) (Hermes et al., 2004; Ludwig et al., 2019). As reported in humans and domestic animals as well as in species in the wild (see above), early pregnancy may provide natural protective mechanisms against premature reproductive senescence. Prolonged non-reproductive periods during long-term maintenance of female rhinoceroses (African and Asian species) and elephants in captivity, are associated with an asymmetric reproductive aging process and subsequent development of genital pathology (leiomyoma) as well as premature senescence (Hermes et al., 2004).

Importantly, issues mentioned above can potentially be overcome by species-specific Assisted Reproductive Technologies to ensure early breeding and fertility extension. Furthermore, the extent of the reproductive life in females can be documented opportunistically when the reproductive age limit is not known (which is the case in most species). A good illustration is the recent birth of a giant panda (*Ailuropoda melanoleuca*) cub by artificial insemination with frozen-thawed semen in one of the oldest females in captivity (Comizzoli, 2020). While artificial insemination was primarily used for management purpose, it ended up provided new data about reproductive aging limit in that species.

As in livestock species and humans, recent studies on cheetahs demonstrate that the age-associated decline in AMH is variable but needs to be taken into consideration to optimize fertility management and decisions about assisted reproduction (Place et al., 2017). Interestingly in cheetahs (*Acinonyx jubatus*), uterine environment in aging females is the real issue while oocyte quality is not affected (Crosier et al., 2011). This can be circumvented by performing *in vitro* fertilization (IVF) on oocytes from old donors and transfer the resulting embryos into young recipient females (Crosier et al., 2020).

## Pending Questions and Next Steps

As highlighted in Table 1, there are many unknowns in wild mammals about the effect of aging on hormonal levels, gamete number and quality, conception, preimplantation embryo, embryo implantation, fetal development, birth, lactation, maternal care, and future fertility of the offspring. Marsupials are not even mentioned in that table because of the absence of data; with the exception being the semelparous marsupial mice (*Antechinus sp.*) that quickly dies after the breeding season (Cohen, 2018). Except for few reports on reproductive aging in rhinoceroses, wild perissodactyls also have not been studied extensively.

As in humans, we need more research in wild species about major cellular processes that are involved in the loss of oocyte quality with advancing age (Quesada-Candela et al., 2021). Except in one study in cheetahs (*Acinonyx jubatus*), nothing is known about the influence of aging in gamete quality (success of fertilization and early embryo development) or uterine environment (Crosier et al., 2011; Ludwig et al., 2019). Even though spermatogenesis or mating behavior does not seem to be impacted by aging in most mammals, there is still a lack of knowledge. There also is no information about reproductive life of offspring born from aging parents (including epigenetic transmissions due to aging).

In non-mammalians species (Table 2), even more research gaps to have to be filled regarding the aging effect on hormonal levels, gamete number and quality, conception, embryo development, metamorphosis, hatching, maternal care, fertility of the offspring. For instance, there are no clear data in amphibians (Jones et al., 2014). There also is a need for more investigations in reptiles that seem to be unique because of the apparent lack of decline in fertility (Hoekstra et al., 2019).

Overall, a clear understanding of the evolutionary causes and consequences of reproductive senescence is still lacking and requires new and integrative approaches. Importantly, investigations of the role of environmental conditions on reproductive senescence also are missing. The epigenetics aspect of reproductive aging needs to be expanded to wild species following examples of research in laboratory and domestic models (Ge et al., 2015; Marshall and Rivera, 2018; Chamani and Keefe, 2019). In addition, the role of miRNAs as marker of reproductive aging (as reported in human ovaries; Battaglia et al., 2020) should also explored.

Although studies in reproductive aging are less advanced in wild species, it can potentially contribute to the general knowledge of a crucial phenomenon and provide new ideas to extend the length and quality of reproductive life in conservation breeding as well as in human reproductive medicine. To fill the current gaps, several options are possible. We first need to better understand the mechanisms behind commonalities and differences observed between species. Findings will then be translatable to other species. There also is the additional value of studying wild species in their natural habitats as sentinel for human populations sharing the same environment. Original aging traits (or non-aging traits) reported above could suggest some research areas or questions in humans, for instance:

• Understanding rapid vs. absence of decline in fertility.
• Exploring sustained oocyte quality in felids (and other species having induced ovulations).
• Examining the influence of parental age on offspring reproductive health (using massive database like the Zoological Management Information System).
• Characterizing the impact of allostatic load (the sum or accumulation of the “burden” due to environment and other stressors like disease, etc. Comizzoli et al., 2019) on reproductive aging.
• Studying more animal models (mammalian and non-mammalian species) known for very long lifespan.
• Using the systems biology approach to better understand and mitigate reproductive aging.

Regarding mitigations and treatments, expanding reproductive life could be achieved through, hormonal treatments (therapies for aging stallions; Turner, 2019), or supplementations during IVF and embryo development for aging donors. Another option would be to increase the ovarian reserve during fetal development (through Bone Morphogenetic Proteins for instance; Regan et al., 2018).

Importantly, lessons learned from human reproductive medicine could also inspire strategies in wildlife. For instance, it would be interesting to explore the impact of aging on reproductive microbiomes (Murphy et al., 2019) since this area is still largely unexplored in wildlife (Comizzoli and Power, 2019). Other examples pertain to the improvement of IVF in aged patients (Gleicher et al., 2016) or the rejuvenation of reproductive tissues through mitochondria biogenesis (Kim et al., 2021).

## Conclusion

Reproductive aging in wild animal species has been mainly documented at the level of overall reproductive senescence rather than at the germ cell development mechanisms. There is a broad consensus about the need to fill research gaps since a lot of cellular and molecular characterizations behind reproductive aging observations remain to be done. There also is a need for more research in male aging across all species. More studies on reproductive aging of target species in their natural habitat (sentinel species) also are crucial to define more accurate reproductive metrics that are relevant to species, including humans, sharing the same habitats. We must keep building bridges between the ecology of senescence and areas of research in reproductive science. Repeated observations in zoo animals also will remain invaluable sources of information to decipher the mystery of reproductive aging.

In sum, wild species can potentially contribute to the general knowledge of a crucial phenomenon. Comparative models will help to understand the fundamental mechanisms underlying reproductive aging and then develop new approaches to extend the length and the quality of reproductive lifespan, which will ultimately impact overall health.

## Author Contributions

PC and MA contributed equally to the preparation and the writing of the manuscript. Both authors contributed to the article and approved the submitted version.

## Conflict of Interest

The authors declare that the research was conducted in the absence of any commercial or financial relationships that could be construed as a potential conflict of interest.
